# Nursing students’ perceptions of clinical learning opportunities and competence in administration of oral medication in the Western Cape

**DOI:** 10.4102/curationis.v43i1.2044

**Published:** 2020-02-19

**Authors:** John J. Musafiri, Felicity Daniels

**Affiliations:** 1 School of Nursing, University of the Western Cape, Cape Town, South Africa

**Keywords:** Bachelor of Nursing, clinical learning opportunities, competence, oral medication, mental health

## Abstract

**Background:**

Medication errors may result in patients’ harm and even death. The improvement of nursing students’ competence in the administration of medication through education and training can contribute to the reduction of medication errors.

**Objectives:**

This study aimed at describing the Bachelor of Nursing students’ perceptions about clinical learning opportunities and competence in the administration of oral medication.

**Method:**

A quantitative descriptive design was employed. An all-inclusive sample of 176 nursing students registered at a university in the Western Cape, South Africa, in 2014 was considered for the study, of whom 125 students consented to participate and completed the questionnaires. Statistical Package for the Social Sciences (SPSS) version 22 was used for data analysis and descriptive statistics were conducted.

**Results:**

The findings showed that a minority of students did not have opportunities to rotate in all specific types of wards. The findings indicated that a total of 92% (115) and 86.4% (108) of the 125 respondents were placed in medical and surgical wards, respectively, where they more likely had opportunities to practise the administration of oral medication. However, 59.2% (74) did not practise administration of oral medication on a daily basis. Only 19.2% (24) of respondents perceived themselves as competent in the administration of oral medication.

**Conclusion:**

The findings indicated that many students perceived their education and training as not providing sufficient learning opportunities to practise the administration of oral medication, whilst the majority of respondents perceived themselves as competent in some of the aspects related to the administration of oral medication, and very few perceived themselves as competent overall in the administration of oral medication.

## Introduction

Medication errors remain a major concern in hospitals worldwide and contribute to the patients’ severe harm and mortality (Tshiamo et al. 2015). Nurses, amongst other healthcare professionals, still make medication errors (Cheragi et al. 2013) and nursing students are also prone to make medication errors (Asensi-Vicente, Jiménez-Ruiz & Vizcaya-Moreno 2018; Cebeci et al. 2015; Keshk & Mersal 2017; Reid-Searl, Moxham & Happell 2010). Several studies have investigated the causes of medication errors by nurses and reported that the main causes are high workload and fatigue caused by extra work, recurrent interruptions during the process of administration of medication, a non-conducive environment for medication administration preparation and non-compliance to medication administration guidelines (Alomari et al. 2018; Cebeci et al. 2015; Hayes et al. 2018; Zarea et al. 2018). According to Cloete (2015), one-third of medication errors happen during administration by nurses. Oral administration of medication is the most common method amongst other routes of medication administration (Liu et al. 2014). Patients prefer to take medication orally (Roy & Prabhakar 2010) because it is likely convenient, painless and non-invasive (Donaldson, Gizzarelli & Chanpong 2007). Given the high occurrence of oral medication prescription, continuing professional development of practicing nurses – who are responsible for medication administration – is needed to improve competence in the administration of oral medication. This will correspond with the findings of studies such as the one conducted in Australia by Aggar and Dawson (2014) that found nursing students lack competence in the administration of oral medication.

In South Africa, studies addressing medication errors made by nurses and nursing students seem to be limited. However, the South African Nursing Council (SANC) disciplined 105 professional nurses who made medication errors between 2003 and 2008 (South African Nursing Council 2012). Kee, Hayes and Macuistion (2009) suggest that medication errors are preventable at the ward level through the application of medication administration guidelines for nurses and nursing students responsible for medication administration. However, it must be acknowledged that the causes of medication errors are broader than personal, environmental and practice-related causes. At nursing education level, the curricula include clinical preparation of nursing students with regard to the administration of medication, therefore making education and practice equally responsible for developing nursing students’ competence in this area (Zare, Purfarzad & Adib-Hajbahery 2013). Opportunities to practise the administration of oral medication in clinical settings, to meet the learning outcomes of a nursing programme, must be purposely planned for nursing students. Henderson et al. (2012) suggest that these learning opportunities are effective if nursing students are integrated into ward activities. Bothma, in Bruce and Klopper (2017) supports this by suggesting that students need to be immersed in life-like experiences and engaged in real-life experiences. This would require that nurse educators and staff in clinical practice engage in a collaborative way to ensure that students have a positive learning experience. However, nursing educators seem to experience challenges regarding the facilitation through the use of didactic content and practical enhancement that will ensure the students gain the knowledge and clinical experiences necessary to administer medication safely (Cooper 2014).

In the School of Nursing where this study was undertaken, the Bachelor of Nursing students have learning opportunities in a general hospital during their second year of study to practise and develop competence in the administration of oral medication. A challenge, the consequence of clinical facilities in the Western Cape accommodating large numbers of students from different nursing schools for clinical learning, is the competition for learning opportunities. Moreover, students do not get enough time to labour on one competency as they are expected to move on to other core competencies in which second year students must prove competence. According to Benner (1984), individuals move from novice to expert in clinical nursing practice, which requires the availability of learning opportunities to develop competence and confidence over time. Based on this notion, students in this programme are placed in a general hospital again during their fourth year of study for consolidation of their learning which includes the administration of medication. Given the reports of medication errors and the time lapse between the second year and final year of study in the Bachelor of Nursing programme, this study investigated students’ perceptions about clinical learning opportunities and their competence in the administration of oral medication at the fourth year level before being registered as professional nurses.

## Literature review

### Medication errors

Several studies, such as the study conducted in Iran by Cheragi et al. (2013), have investigated the causes of medication administration errors. This study included 237 nurses and reported that the common causes of medication administration errors were the lack of pharmacological knowledge, shortage of nurses compared to the number of patients who received medication and the use of medication abbreviations and synonyms. Other common causes of medication administration errors, such as illegibility of medication prescriptions, fatigue because of the workload and the use of lookalike and sound-alike medications, were reported by a study conducted on 423 nurses in Iran by Izadpanah et al. (2018) and a study conducted on 965 nurses in Serbia by Svitlica, Simin and Milutinovi (2017). A study amongst nursing students in Turkey conducted by Cebeci et al. (2015) reported that the incompetence in performing the administration of medication and medication errors was linked to the high workload in the ward and stress and fatigue.

Studies have shown that nursing students’ knowledge and competence in medication administration is lacking (Mariani et al. 2017; Sulosaari et al. 2015). In a study conducted in Belgium by Dilles et al. (2011), nursing students reported that they did not perceive themselves as able to safely administer medication. These students are therefore at risk of making medication errors. In this regard, a study conducted in Iran by Vaismoradi et al. (2014) reported that all the nursing student respondents perceived that their nursing programme left them vulnerable to medication errors. This report is supported by a study conducted in Turkey by Cebeci et al. (2015), which found that a total of 38.3% (124) of the 324 nursing students who participated made medication errors. Similarly, a study conducted in the Philippines by Apsay et al. (2018) using 388 nursing students recruited from the third year and fourth year levels of a nursing programme found that 17.3% of the respondents made medication errors. In another study conducted in Indonesia by Musharyanti et al. (2019), 26 nursing students who were interviewed stated that they administered medication at the wrong time and/or failed to identify the patient prior to medication administration. Nursing student participants in the study by Musharyanti et al. (2019) stated that the lack of knowledge and skills, the lack of good role models and proper supervision contributed to the causes of the medication administration errors. Given the involvement of nursing students in medication errors, nursing schools and clinical supervisors are required to provide extensive support in facilitating learning for students who are expected to become competent and safe practitioners (Vaismoradi et al. 2014). This is supported by the South African Nursing Council (2013b) which requires the nursing school’s accountability with regard to clinical supervision.

### Clinical learning opportunities

There is a lack of studies in South Africa focusing on administration of oral medication, particularly related to students’ clinical placement, orientation, allocation of duties and supervision in specific clinical settings. Clinical placement forms a key component of nursing education and it is considered as crucial as it helps the students perceive the reality of nursing and gain experience (Hilli, Salmu & Jonsén 2014). However, clinical placements must be relevant to the student’s learning outcomes. This view is supported in a study conducted in Canada by Hartigan-Rogers et al. (2007), where nursing students indicated a clinical placement preference for medical-surgical wards in which they could get more opportunities to practise their skills. The SANC emphasises the value of clinical learning to enhance the experience of nursing students (South African Nursing Council 2013a). In this regard, nursing students administer medication under supervision to enhance their skills in medication administration (Apsay et al. 2018). Clinical practice therefore allows students to gain nursing skills prior to becoming professional nurses (Gemuhay et al. 2019). Improvement of clinical learning opportunities in the administration of oral medication might therefore enhance the competence of nursing students in this skill and reduce medication administration errors (Betts 2016). However, little is known about studies that investigated the clinical learning opportunities that were available to nursing students in specific wards. A study conducted in Iran by Baraz, Memarian and Vanaki (2015), for example, indicated that nursing students complained about the lack of clinical learning opportunities in orthopaedic, surgical and gastroenterology wards though they did not specify the types of opportunities that were scarce.

According to their scope of practice, the administration of medication by nursing students should be conducted under the supervision of a registered nurse (South African Nursing Council 2013b). In this regard, Jeggels, Traut and Africa (2010) argue, whilst suggesting that clinical supervision plays a significant role in the development of clinical skills of Bachelor of Nursing students, that the professional nurse working in the ward is the supervisor of these students. The majority of the 276 nursing students who participated in a study conducted in Finland by Helminen, Tossavainen and Turunen (2014) reported a positive experience regarding supervision; students’ appreciation of sufficient time spent with their clinical supervisors in their clinical practice was highlighted. Meaningful clinical learning and supervision therefore seem to positively influence nursing students’ learning (Reid-Searl et al. 2010), which would include the administration of oral medication.

### Competence in administration of oral medication

Clinical supervisors assess nursing students’ competence through the use of an observation check as used in the United States (Cant, McKenna & Cooper 2013); this assessment method is mostly applied to the administration of oral medication. These students, however, should be given an opportunity to assess their competence in this regard. In support of this statement, a study conducted in Norway by Dale, Leland and Dale (2013) suggested that nursing students have the responsibility to conduct self-assessment of their level of competence. The practice and assessment of oral medication administration requires the adherence to guidelines such as the identification of the prescription chart and the patient, identification of medication preparation, check and recheck of oral medication (Downie et al. 2008; Perry, Potter & Elkin 2012). In this regard, the principle of ‘five rights’, emphasised by Lin et al. (2014) and commonly referred to as the golden rules, should be observed throughout the procedure of medication administration. The five golden rules include the identification of the patient, to ensure that he or she is the right patient; identification and administration of the right drug; checking and administration of right dosage; checking the right time; and right route such as the oral route of medication administration applied in this study. Moreover, the patient’s safety is a priority throughout the medication administration procedure (Elliott & Liu 2010).

The SANC disciplined nine nursing students who were involved in medication errors between 2003 and 2008 (South African Nursing Council 2012). However, there is no evidence that all medication errors made by nursing students were reported to SANC or hospital nursing management for corrective measures. Given the reports of medication errors and that the nature of nursing education programmes seems to pose challenges for students to become competent in the administration of oral medication, understanding the students’ perceptions about their clinical learning opportunities and competence is important, yet unknown in South Africa.

### Problem statement

Nursing students at a university in the Western Cape province of South Africa are expected to be competent in the administration of oral medication at the end of the second year level of the Bachelor of Nursing programme. However, the nature of nursing education programmes seems to pose challenges for students to become competent in the administration of oral medication. Although during their fourth year level of study, the students are placed in a general hospital for consolidation of the skills learnt in their second year level, the clinical learning programme of the third and fourth years might not allow the students to continue the practice of oral medication administration and achieve the level of competence as described by Benner (1984). In this regard, the students’ competence in oral medication administration appears questionable at the fourth year of nursing programme prior to registration as professional nurses. Currently, medication errors are a problem and Jevon et al. (2013) argued that the improvement of the skills and the competence of nursing students through the training can contribute to the reduction of these errors. However, the students’ perceptions about clinical learning opportunities and their competence in the administration of oral medication are unknown.

### Significance of the study

The findings of this study will inform the School of Nursing about the adequacy of clinical learning and competence of the students in the administration of oral medication. Moreover, the study will contribute to improve the alignment of clinical learning with the learning outcomes of the Bachelor of Nursing programme. In this regard, patients will benefit from this study as the improved training of the students will contribute to the reduction of medication errors.

### Definitions of the key concepts

*Bachelor of Nursing* is a degree awarded to a nursing student who has successfully completed four academic years of a nursing curriculum (South African Nursing Council 1988).

*Clinical learning opportunities* refer to (South African Nursing Council 2013b:5):

[*T*]he range of learning experiences, including work-integrated and service learning, available in a healthcare setting, which may also include other experiential learning sites where a learner has the opportunity to gain the required clinical skills. (p. 5)

In this study, clinical learning opportunities refer to the opportunities which students have during a clinical placement within a general hospital with regard to orientation, allocation of the task, supervision and practice of administration of oral medication.

*Competence* refers to ‘the ability of a practitioner to integrate the professional attributes’ (South African Nursing Council 2013b:2). In this study, competence refers to the degree of performance of all competencies or skills related to the administration of oral medication.

*Oral medication*, in this study, refers to medication administered by a nursing student via the mouth of the patient admitted in general hospital.

*Clinical placement*, in this study, refers to a period of time that the second-year Bachelor of Nursing students registered at the School of Nursing spend in the ward within a general hospital. During this time, the students are exposed to patient care and learning of nursing skills through the integration of theory and practice.

*Supervisor*, in this study, refers to a professional nurse who is a clinical educator responsible for verification of students’ attendance in the clinical placement by checking the student’s signed clinical learning hours against the student’s off duty in the ward; he or she is also responsible for the facilitation of student’s clinical learning. The supervisor trains the students through the demonstration of skill, guides them in the completion of a skill and in their professional attitude and assesses the student’s competence in performance of a skill.

### Aim

The study aimed to describe the fourth year Bachelor of Nursing students’ perceptions regarding clinical learning opportunities and their competence in the administration of oral medication at a general hospital.

## Research methodology

### Research design

A quantitative cross-sectional descriptive design (Brink, Van der Walt & Rensburg 2012; Polit & Beck 2012) was used to collect the data at one point in time using Bachelor of Nursing students and describe their perceptions.

### Population and sampling

Population, defined as a group of people or subjects in their entirety who are of interest to the researcher (Brink et al. 2012; Burns & Grove 2011), refers to the Bachelor of Nursing students who are registered at a university in the Western Cape in 2014. Because of the relatively small population (*N* = 176), an all-inclusive sampling (Terre Blanche, Durrheim & Painter 2006) strategy was used, which provided a sample of 176 fourth year Bachelor of Nursing students.

### Data collection method

The self-report questionnaire developed by the researcher was based on the reviewed literature and an observational checklist borrowed, with permission, from Zare et al. (2013) which was used in Iran. The questionnaire was reviewed and found to be valid by the study supervisor and the results of a pre-test that involved 22 participants. Moreover, Cronbach’s alpha value was 0.979; therefore, the self-report questionnaire consisting of 90 closed-ended, mostly five-point Likert scale-type questions was reliable.

Rigour, in terms of validity and reliability, was ensured. The researcher obtained an alphabetical list of the students and booked a suitable venue where the data would be collected. One week before the collection of data, the researcher invited all the 176 students through their e-mails to participate in the study and the participant information sheet was sent to them. The students were informed that the data would be collected on 30 November 2014; time and venue were also communicated. On the day of data collection, the researcher explained the purpose of the study to the participants who were allowed to ask questions. All the ethical aspects were explained to the participants (Grove, Burns & Gray 2013). A total of 125 students consented in writing to participate in the study and completed a self-report questionnaire which took approximately 30 min. The researcher collected the consent forms and the self-report questionnaires that were kept separately in a safe and lockable place to protect the information and identity of the participants.

### Data analysis

A check was conducted by the researcher to determine whether all the self-report questionnaires were legible and complete. According to Babbie (2010) who argued that the researcher handles the quantitative analysis by the computer programs, in this study the researcher entered the data into the computer program to quantify the data. A number (code) was assigned to each participant’s questionnaire to allow easy identification by the researcher. Data were analysed using Statistical Package for the Social Sciences version 22. Descriptive, univariate and bivariate analyses were conducted. The Bartlet test of sphericity which is significant at 0.05 for factor analysis to be appropriate was significant at 0.00 for all variables.

### Ethical considerations

All the principles of research ethics were applied. The study was approved by the Senate Research Committee of the University of the Western Cape (registration number 14/6/24).

## Findings and discussion

### Clinical learning opportunities

According to the School of Nursing included in this study, clinical placement for the second year nursing students was on a rotational basis through medical, surgical, paediatric, orthopaedic, theatre, trauma, urology, neurology wards as well as gynaecology and dermatology wards within the general hospital. However, the results of this study indicate that 125 respondents did not have equal opportunities to rotate through these wards. Each student was however placed in more than one ward (see Table 1). It is a requirement from the South African Nursing Council (1985) that each student is placed in a general hospital and is rotated through medical, surgical, paediatric, theatre, trauma and other wards which the School of Nursing must specify based on the accreditation of clinical learning sites for the programme. The results showed that the majority of 125 respondents were placed in a medical ward (92%, 115) and a surgical ward (86.4%, 108), where they could have more opportunities to practise administration of oral medication. Table 1 shows more results regarding the students’ clinical placement opportunities according to their rotation in different wards. The students placed in the top four wards (see Table 1) during the second year or fourth year of their study would most likely have been more exposed to the administration of oral medication than the students who were not placed in these wards. For example, students placed in theatre or trauma ward would not necessarily have been exposed to the administration of oral medication as the routine of these wards differs from that of other wards. At best, the students in a trauma ward might be required to administer analgesics to the patients. The study, however, did not establish how many students had exposure to more than one of the wards where they would have been more likely to practise the administration of oral medication or how many students did not have exposure to these wards. Similar to this study, a study conducted in Australia by Reid-Searl et al. (2013) found that a higher number of student respondents placed in a medical ward (62%) and surgical ward (76%) had opportunities to practise the administration of medication than those who were not placed in such wards. In contrast to these findings, a study conducted in Finland by Kajander-Unkuri et al. (2014) found that 35% of the 154 nursing student participants were placed in theatre or a surgical ward and only 19% were placed in a medical ward. As mentioned earlier, the alignment of theory and practice is imperative for meaningful learning. It is, however, unclear whether the placement of students in these studies was relevant to their learning outcomes.

**TABLE 1:** Respondents’ clinical placement opportunities.

Clinical placement	*n* = 125	%
Medical ward	115	92.0
Surgical ward	108	86.4
Paediatric ward	70	56.0
Orthopaedic ward	61	48.8
Theatre ward	59	47.2
Trauma ward	58	46.4
Urology ward	28	22.4
Neurology ward	27	21.6
Gynaecology ward	26	20.8
Dermatology	12	9.6

Furthermore, the students expected to be orientated during their clinical placement especially with regard to the competencies that were part of their expected clinical learning outcomes. In this regard, a total of 24.8% (31) participants strongly agreed and 44.8% (56) agreed that they were orientated to ward routines in the administration of oral medication. The results also showed that a total of 60.8% (76) of participants were orientated by a professional nurse on duty in the ward. In this regard, the researcher also had to establish whether the professional nurse-in-charge of the ward was informed about their learning needs. Of the 125 respondents, 20.8% (26) strongly agreed and 43.2% (54) agreed that the professional nurse-in-charge was informed. Furthermore, the results of this study indicated that the majority of the students (89.6%, 112) were orientated by a clinical supervisor who was able to align the practice with theory.

A cumulative total of 20% (25) of the 125 respondents strongly agreed and agreed that they were allocated to administer oral medication on a daily basis. Overall, this minority of the 125 respondents would have been able to develop their level of competence and confidence therein. As students do not enjoy supernumerary status in the ward, they are allocated to other patient care activities as required in the ward. Students are often challenged by the fact that clinical competencies are performed differently in practice than in the skills laboratory. The findings of this study indicated that a total of 28.8% (36) of respondents strongly agreed and 47.2% (59) agreed that they had the opportunity to administer many types of oral medication at one time to the same patient. With respect to an opportunity to administer oral medication to all the patients in the ward during a medication round, a total of 24.8% (31) of respondents strongly agreed and 40.8% (51) agreed that they had an opportunity. Of the 125 respondents, 30.4 % (38) strongly agreed and 37.6% (47) agreed that they had more than two practice opportunities to administer oral medication. Aggar and Dawson (2014) affirmed that achievement of skills and competence in the administration of oral medication is a challenge for nursing students. Therefore, nursing students who did not practise this competency might encounter problems after registration as a professional nurse and stand the risk of making medication errors. Despite the minority of respondents not having opportunities to practise the administration of oral medication, this still goes against the SANC requirements (South African Nursing Council 1985) and is not supported by the experiential learning and theoretical underpinning of clinical learning in the Bachelor of Nursing programme.

Clinical supervisors who are experienced professional nurses must ensure that nursing students meet the clinical learning outcomes regarding the practice of oral medication administration, which is currently not occurring. The study findings revealed that the minority (30.4%, 38) of the 125 respondents were mostly supervised by the clinical supervisor, who was skilled in aligning clinical learning with the theory learnt in the classroom. With regard to engaging in a practical session in the presence of the supervisor prior to clinical assessment, 8.8% (11) of respondents collectively strongly disagreed and disagreed, whilst 9.6% (12) were uncertain that this occurred; 38.4% (48) of respondents strongly agreed and 43.2% (54) agreed that they had a practice session. Regarding the feedback from the clinical supervisor on their performance, a total of 38.4 % (48) of respondents agreed and 48.8% (61) strongly agreed that they received feedback. A combined total of 87.2 % (109) of the 125 respondents strongly agreed and agreed that they were assessed on one patient in order to be found competent. This study did not investigate students who were not supervised by the clinical supervisor during administration of oral medication. It would however be concerning if students are not guided by a clinical supervisor in the administration of oral medication. This challenge was confirmed in the study conducted in Cape Town by Klerk (2010), which found that clinical supervisors were not visible in the clinical environment. Clinical supervision roles seem blurred – Rikhotso, Williams and De Wet (2014) argued that it is unclear who is responsible for clinical supervision of nursing students within South Africa. As the clinical supervision task is not limited to the clinical supervisors only, a total of 38.4% (48) of the 125 respondents administered oral medication mostly under the supervision of a professional nurse on duty in the ward. In this regard, Jeggels et al. (2010) argued that a professional nurse on duty in the ward is a supervisor of nursing students. Surprisingly, a total of 24% (30) of the 125 respondents were mostly supervised by an enrolled nurse. Again this blurring was evident in students reporting that they were supervised by an enrolled nurse and a senior nursing student in the ward, which is of concern especially when the competence level of the mentor is uncertain. The level and quality of support and guidance provided by enrolled nurses and fourth-year nursing students, however, were not established in this study.

The findings of this study showed that 68.8% (86) of respondents confirmed that the practice related to the administration of oral medication was aligned with the theory. The importance of the alignment of clinical teaching, by nursing schools, to correct and current practice cannot be over-emphasised. However, it is not always the case that competencies are practised correctly in the clinical setting. This is supported by the study conducted in Australia by Reid-Searl et al. (2013) using 45 nursing students of whom a collective total of 42% of student respondents strongly disagreed, disagreed or were not sure that the principle of medication administration was respected by professional nurses. For instance, the findings of the current study indicated that a total of 40.8% (51) strongly agreed and 44.8% (56) of the 125 respondents agreed that they practised handwashing before preparation of medication. However, for washing and disinfecting of their hands between patients, 25.6% (32) of respondents strongly agreed and 29.6% (37) agreed that they adhered to this practice. Regarding the cleaning practice, a cumulative total of 68% (85) strongly agreed and agreed that they cleaned the medication trolley.

### Perceived competence in administration of oral medication

The items in Table 2 are presented in three sections: the first section is related to medication trolley preparation and checking of medication, the second section is associated with patient involvement and medication administration, whilst the third section is related to record-keeping and medication storage. These items are ranked from the highest to the lowest scores in each section, based on the cumulative scores of the options ‘agree’ and ‘strongly agree’ for each of the 42 listed skills. A student was regarded as competent in the 42 skills listed in Table 2 when he or she selected the options ‘agree’ or ‘strongly agree’. It is expected that students should be competent in all 42 skills included in the questionnaire to avoid medication errors. However, according to the School of Nursing included in this study, a student is competent in the administration of oral medication when he or she performs each critical skill at 50% or more and obtains the final mark of 50% and above (School of Nursing 2019). However, the findings of this study indicated that only a total of 19.2% (24) of the 125 respondents were competent in all 42 skills listed. Lin et al. (2014) argued that nurses have traditionally been applying the principle of ‘five rights’, which includes the right patient, right drug, right time, right route and right dose. Based on these rights, which are commonly called the five golden rules, the findings of this study indicated that the majority of the respondents were competent in applying this principle during administration of oral medication.

**TABLE 2:** Perceived competence in medication administration.

Items	Agree		Strongly agree		Total (*n* = 125)		Mean	SD
	*n*	%	*n*	%	*n*	%		
**Medication trolley preparation and medication check**								
Checking time and frequency of medication on prescription chart	52	41.6	68	54.4	120	96.0	4.49	0.64
Checking doctor’s prescription	55	44.0	64	51.2	119	95.2	4.44	0.67
Checking the name of medication on prescription chart	45	36.0	74	59.2	119	95.2	4.51	0.70
Checking the route of medication administration on prescription chart	40	32.0	78	62.4	118	94.4	4.54	0.69
Checking medication dose on prescription chart	43	34.4	74	59.2	117	93.6	4.51	0.67
Checking previous time of medication administration	54	43.2	61	48.8	115	92.0	4.40	0.66
Checking the expiry date of medication	46	36.8	60	48.0	106	84.8	4.25	0.92
Checking for indication of allergy on prescription chart	50	40.0	55	44.0	105	84.0	4.19	0.93
Washing hands before setting medication trolley	60	48.0	44	35.2	104	83.2	4.11	0.89
Taking the container of medication against the prescription chart	42	33.6	60	48.0	102	81.6	4.17	1.03
Identifying scheduled drug if applicable	57	45.6	45	36.0	102	81.6	4.11	0.86
Checking medication a second time against the prescription chart	43	34.4	59	47.2	102	81.6	4.18	0.98
Identifying the alternative name of the medication if needed	61	48.8	38	30.4	99	79.2	3.99	0.94
Checking the name and signature of medical practitioner	41	32.8	58	46.4	99	79.2	4.14	1.02
Cleaning used items	52	41.6	40	32.0	92	73.6	3.92	1.02
Cleaning medication trolley	54	43.2	37	29.6	91	72.8	3.85	1.09
Checking the side-effects	44	35.2	28	22.4	72	57.6	3.56	1.11
Checking drug interaction, e.g. with/before/after meals	38	30.4	24	19.2	62	49.6	3.42	1.10
**Patient involvement and medication administration**								
Taking the correct medication prior to be given	48	38.4	72	57.6	120	96.0	4.51	0.66
Administration of correct dose to the patient	51	40.8	68	54.4	119	95.2	4.46	0.73
Offering of water to the patient	52	41.6	66	52.8	118	94.4	4.42	0.78
Calculation of correct dose of medication	49	39.2	65	52.0	114	91.2	4.41	0.73
Reporting abnormalities to the sister-in-charge	50	40.0	62	49.2	112	89.2	4.36	0.76
Ensuring patient swallows the medication	46	36.8	63	50.4	109	87.2	4.30	0.92
Making the patient comfortable	58	46.4	48	38.4	106	84.4	4.15	0.89
Ensuring the patient’s safety, for example, bed cots raised and bell in reach of patient	57	45.6	47	37.6	104	83.2	4.14	0.86
Identification of prescription chart by checking patient’s name against patient’s identification band	43	34.4	57	45.6	100	80.0	4.12	1.06
Giving health education to the patient	56	44.8	41	32.8	97	77.6	4.03	0.89
Checking contra-indications, for example, NPO, nausea, HB, HGT and bradycardia	46	36.8	47	37.6	93	74.4	4.03	0.95
Monitoring any immediate side-effect of medication	49	39.2	41	32.8	90	72.0	3.92	1.02
Obtaining verbal consent from the patient	47	37.6	42	33.6	89	71.2	3.90	1.05
Checking the diagnosis of patient	42	33.6	42	33.6	84	67.2	3.86	1.08
Explaining the procedure to the patient	45	36.0	39	31.2	84	67.2	3.79	1.13
Assessing the patient’s basic needs	45	36.0	33	26.4	78	62.4	3.73	1.05
Checking the vital signs of the patient	33	26.4	37	29.6	70	56.0	3.68	1.09
**Record-keeping and medication storage**								
Signing the prescription chart in the correct block	48	38.4	69	55.2	117	93.6	4.44	0.77
Locking medication trolley after ruse	47	37.6	69	55.2	116	92.8	4.45	0.73
Recording the scheduled drugs in scheduled drug book	48	38.4	67	53.6	115	92.0	4.43	0.72
Checking the balance of scheduled drug under supervision of a professional nurse	60	48.0	51	40.8	111	88.8	4.25	0.79
Explain the role of witness to countersign for administered scheduled drug, if applicable	44	35.2	59	47.2	103	82.4	4.20	0.98
Explaining the safe-keeping of the scheduled drug cupboard key, if applicable	38	30.4	58	46.4	96	76.8	4.15	0.98
Explaining the role of witness before administration of scheduled drug, if applicable	54	43.2	32	25.6	86	68.8	3.75	1.09

NPO, nothing per mouth; HB, Hemoglobin; HGT, Hemo gluclose test.

Regarding the identification of the right patient, the current study’s findings indicated that 80% (100) perceived themselves as competent in checking patients’ names against their identification bands; however, Schneidereith (2014) argued that nursing students are ‘lazy’ to adhere to the principle of the administration of medication. Pertaining to the right drug, the results indicated that a total of 95.2% (119) of the 125 respondents were competent in checking the medication name on the prescription chart. Furthermore, a total of 81.6% (102) were competent in checking the medication container against the patient’s prescription chart to match the medication name to the patient’s name. Comparative to these findings, in a study conducted in Korea by Kim and Bates (2013), a total of 98.6% of participants checked the medication name at least once. Failure to check the medication name on the prescription chart may lead to the administration of wrong medication. In support of this statement, a study conducted in South Africa by Labuschagne et al (2011) found that 48.6% of the respondents administered the wrong medication. The majority (94.4%, 118) of the 125 respondents in this study reported competence in checking the route of medication on the prescription chart, meaning that the majority of the respondents adhered to the right route.

The majority of the 125 respondents affirmed that they were competent in checking the time and frequency of medication on prescription chart (96.0%, 120) and in checking the previous time of medication administration (92.0%, 115). It is crucial to comply with the frequency at which medication is prescribed by the doctor, to prevent medication overdose or missing a dosage (Perry et al. 2012). In this regard, a study conducted in Ethiopia by Agalu et al. (2012) indicated that 15.5% of medication errors were because of the medication being given at the wrong frequency. Similarly, a study conducted in the Netherlands by Van den Bemt et al. (2009) indicated that 18% (77) of 428 medication errors were related to the wrong time of medication administration. In a study conducted in Korea by Kim and Bates (2013), a total of 41.0% of participants administered the medication at the correct time. A total of 95.2% (119) of participants were competent in the administration of the correct dose. Table 2 presents more results. Administration of oral medication involves record-keeping (Elliott & Liu 2010); the majority of the 125 respondents were competent in this regard (see Table 2). The presence of a signature on the prescription chart indicates that the medication was administered. This prevents the repeated administration of medication, which can lead to a possible overdose. In this study, the majority of respondents were perceived competent in checking the medication dose on the prescription chart (see Table 2). Bearing in mind that nursing students included in this study were in their final year prior to registration with the SANC as professional nurses, the potential risk to the patients posed by the students who did not regard themselves as competent in some skills should not be underestimated in their future nursing practice.

## Limitations

This study is limited to the use of a self-assessment tool which has potential for bias. Therefore, the results regarding the students who perceive themselves competent in the administration of oral medication might not be the true reflection of the students’ competence if they were actually assessed in practice.

## Recommendations

This study recommends that schools of nursing collaborate more closely with staff in clinical practice around designing clinical learning for nursing students. To enhance the competence of Bachelor of Nursing students in future and reduce medication errors, which contribute to the patients’ harm and death, an increase in clinical learning opportunities in medication rounds under supervision of professional nurses and an increased use of simulation related to administration of oral medication in the skills laboratory are recommended. It is recommended that the School of Nursing and the general hospital’s management give equal opportunities to all nursing students to rotate in different types of wards as required by the South African Nursing Council (1985). It is further recommended that researchers investigate the nature and extent of medication errors by nursing students in South Africa to inform remediation, as this is unknown.

## Conclusion

The findings of this study highlighted several areas of improvement with regard to students’ clinical learning opportunities; the involvement of both the nursing school and clinical facilities is needed to improve the practice of oral medication administration. The findings show that the majority of respondents perceived themselves as competent in some aspects related to the administration of oral medication;– however, few perceived themselves as generally competent in this competency.
